# Identifying volunteer groups based on motivational patterns using latent class analysis

**DOI:** 10.3389/fpubh.2026.1836217

**Published:** 2026-07-16

**Authors:** Harrid Nkhoma, Samuel Manda, Tapiwa Kamuruko

**Affiliations:** 1 Department of Statistics, University of Pretoria, Pretoria, South Africa; 2 Voluntary Advisory Services Section, United Nations Volunteers (UNV), Bonn, Germany

**Keywords:** latent class analysis, self-determination theory (SDT), sustainable development, volunteer motivation and endorsement, world values survey

## Abstract

**Background:**

Volunteers dedicate their time and effort to enhancing communities, supporting social causes, and fostering social cohesion. Self-Determination Theory suggests that volunteer motivation may vary along a continuum of internalization from controlled to autonomous motivation. However, there is limited understanding of whether motivational profiles reflect qualitatively distinct types or primarily differ in intensity. This study seeks to identify fundamental motivational groupings among volunteers and to analyse the sociodemographic factors associated with these profiles using data from a cross-national survey.

**Methods:**

Data from 2,605 respondents in the World Values Survey (1982–2022) were analysed using 14 indicators of volunteer motivation. Two to six classes were fitted using Latent Class Analysis (LCA). Multinomial logistic regression subsequently examined associations between class membership and sociodemographic characteristics.

**Results:**

A three-class model was identified, characterized primarily by varying degrees of motivational intensity: low (31.2%), moderate (32.1%), and high (36.7%) motivation. Classes showed parallel response patterns across altruistic, moral, social, religious, and self-oriented motivations, suggesting quantitative rather than qualitative differences. Older adults (35–54 years: OR = 0.62; 55 + years: OR = 0.59) and women (OR = 0.67) were less likely to belong to the low-motivation group, while higher education (OR = 1.44) was associated with greater likelihood of low motivation.

**Conclusion:**

Volunteer motivations differ primarily in intensity rather than type, suggesting a continuum of motivational engagement rather than distinct volunteer profiles. Volunteer policies should acknowledge individual differences in motivational strength and provide opportunities designed to foster deeper and more sustained engagement. Considering sociodemographic and cultural differences can help ensure volunteering initiatives are inclusive, sustainable, and effective across diverse global settings.

## 1 Introduction

Volunteers play a vital role in addressing community needs, environmental challenges, and social inequalities, thereby promoting sustainable development and strengthening social cohesion (1). They have spearheaded international efforts to combat climate change and protect the planet through engagement in environmental initiatives such as nature conservation and plastic waste reduction (2). A comprehensive understanding of the motivations driving individuals to volunteer is crucial for formulating effective and inclusive volunteering policies. Individuals contribute their time and skills voluntarily, often for interconnected reasons that do not involve monetary compensation. While it is widely acknowledged that volunteering is influenced by multiple motivational factors (3), there remains limited evidence on the interaction of these motivations and on how policy frameworks can effectively facilitate, sustain, and expand volunteer participation.

Most research has examined volunteer motivations as separate dimensions or independent predictors of volunteering behavior. While these studies have substantially advanced our understanding of why people volunteer, they have generally adopted a variable-centred approach, examining the effects of individual motivations in isolation (3, 33, 34). Research has also shown that socio-demographic and contextual factors, including age, gender, education, employment status, and cultural context, influence individuals’ motivations to volunteer (5, 15, 39). For example, younger individuals may be motivated by opportunities for skill acquisition and career development, whereas older adults are more likely to emphasise social connections, altruistic values, and moral obligations (5). However, these studies have typically examined motivations and their determinants separately, providing limited insight into how multiple motivations coexist within individuals and how socio-demographic and contextual factors shape distinct motivational profiles. Consequently, a person-centred approach is needed to identify latent motivational profiles and better understand the heterogeneity of volunteer motivation.

Volunteer motivation has increasingly been conceptualised within broader motivational theories, particularly Self-Determination Theory (SDT), which distinguishes between autonomous and controlled forms of motivation and views motivation as lying along a continuum rather than as discrete categories (9, 11). From this perspective, volunteering may be driven by a combination of intrinsic enjoyment, internalised values, social obligations, and external incentives, with individuals differing in the strength and degree of internalisation of these motivations (10). Although SDT acknowledges that multiple motivations coexist within individuals, relatively few studies have adopted person-centred analytical approaches to identify motivational profiles that capture this complexity.

To address this gap, this study employed Latent Class Analysis (LCA), a person-centred statistical approach that identifies unobserved subgroups of individuals based on shared response patterns (16, 20). LCA has been increasingly applied in the social and behavioral sciences to identify patterns in attitudes, behaviors, and demographic characteristics (20), and in psychometric research to uncover underlying traits in survey responses, thereby enhancing understanding of constructs such as mental health and social values (21). In health and social behavior research, it has been used to segment populations for targeted interventions and, through approaches such as Latent Transition Analysis, to examine changes in group membership over time (16). In volunteer research, LCA has successfully identified motivation profiles among university students (4) and nursing students during the COVID-19 pandemic (22), demonstrating its value in understanding the heterogeneity of volunteer motivations across populations.

Despite these advances, applications of LCA to volunteer motivation remain limited, particularly in cross-national settings. Most existing studies have focused on specific populations or countries, limiting the generalisability of their findings. Accordingly, this study applies LCA to World Values Survey data to identify latent motivational profiles among volunteers across seven countries. Specifically, the study aims to (i) identify distinct profiles of volunteer motivation, (ii) describe these profiles by their predominant motivational characteristics, and (iii) examine how socio-demographic and country-level factors influence profile membership. By identifying latent motivational profiles, this study provides empirical evidence to inform more targeted volunteer recruitment, improve volunteer retention, and enhance the effectiveness of volunteer programmes. These findings are particularly relevant for organisations seeking to strengthen volunteer engagement and contribute to achieving the Sustainable Development Goals (SDGs).

This study employed Latent Class Analysis, a person-centered approach that identifies subgroups of volunteers characterized by diverse motivational patterns (16, 17). The analysis categorises volunteers by their primary motivations and examines how demographic factors such as age, gender, and country of residence influence group membership (18, 19). LCA is frequently utilised in the social sciences to uncover patterns in attitudes, behaviors, and demographic characteristics (20), as well as in psychometric research to elucidate phenomena such as mental health and social values (21). In the fields of health and social research, LCA facilitates targeted interventions and monitors changes within groups over time (16). Within the context of volunteering research, LCA has been employed to classify individuals based on motivation; for instance, Geiser et al. (4) identified six groups among university students, linking strong internal motives with increased volunteering, while He et al. (22) identified three groups among nursing students during the COVID-19 pandemic, highlighting differences in support and stress levels. Additional studies conducted in Singapore have categorized volunteers according to their satisfaction and incentives (23).

Despite the theoretical insights offered by SDT, empirical research has largely examined volunteer motivations as discrete factors, exploring how altruistic, social, or self-oriented motives independently predict volunteering behavior (3, 12). This variable-centered approach, while valuable, cannot capture how multiple motivations coexist and interact within individuals, nor does it reveal whether volunteers differ qualitatively in their motivational types or quantitatively in motivational intensity (24). A person-centered approach, such as Latent Class Analysis (LCA), offers the opportunity to identify subgroups of volunteers who share similar motivational patterns, thereby revealing the underlying structure of volunteer motivation whether it is best characterized by discrete motivational types or a continuum of motivational intensity (16, 20). This distinction has important theoretical implications: finding qualitatively distinct profiles would support a typological conceptualization of volunteer motivation, while finding primarily intensity-based differences would align with SDT’s continuum perspective. While SDT is primarily concerned with the quality and internalization of motivation, our study examines the intensity and pattern of motivational endorsement across multiple domains.

Despite previous LCA studies having identified motivational subgroups in specific populations (4, 22), these studies have primarily focused on narrow contexts (e.g., university students, nursing students during COVID-19) and have not systematically examined whether identified profiles reflect qualitative or quantitative differences. Moreover, little is known about how motivational profiles vary across culturally diverse national contexts, despite evidence that cultural norms and institutional structures shape volunteer engagement (4, 13). Additionally, sociodemographic factors such as age, gender, education, and employment status have been associated with volunteering behavior (7, 25), but their relationship to motivational profiles rather than just volunteering participation remains underexplored.

By identifying concealed motivational patterns, this research provides empirical evidence to facilitate more targeted recruitment, improve retention rates, and augment the efficacy of volunteer programmes, particularly those aligned with the Sustainable Development Goals (SDGs).

## 2 Methods

### 2.1 Data source and variables analysed

We utilised secondary volunteer data from the World Values Survey (WVS) (1982–2022). The World Values Survey (WVS) is a comprehensive, cross-national household survey designed to assess individuals’ beliefs, values, and social behaviors across diverse cultural contexts in 120 countries. The survey is conducted using standardised procedures in each participating nation, typically through face-to-face interviews or computer-assisted personal interviewing (CAPI), depending on local infrastructure. For this analysis, data were obtained from the most recent available wave. Our study specifically examined volunteer motivation data from seven countries: Argentina, Brazil, Chile, China, Japan, Russia, and Nigeria. The seven countries were selected based on several criteria: (1) representation of diverse geographical and cultural regions (South America: Argentina, Brazil, Chile; East Asia: China, Japan; Africa: Nigeria; Europe/Asia: Russia); (2) availability of complete data on all 14 volunteer motivation indicators; (3) varying levels of volunteer participation rates and cultural attitudes toward volunteering; (4) recent data availability on volunteer motivation variables (1982–2022). Much as this data is from different regions where there is likely to be volunteering measurement variances, no measurement invariance test was conducted because WVS uses standardized questionnaire and survey methodology across participating countries and regions. WVS assumes that the 14 motivational items are applicable across diverse volunteer activities and that the underlying motivational structure is consistent regardless of activity type. This assumption is necessary given the aggregated nature of the WVS data. While the WVS uses standardized procedures across participating countries, we acknowledge that cultural differences in volunteer norms, social desirability, and questionnaire interpretation may affect responses.

In certain iterations of the World Values Surveys, respondents were asked about their volunteering for a predefined list of organisations in the preceding month. For those who responded affirmatively, a series of items was employed to assess the significance of each of the 14 motivations or reasons, rated on a five-point Likert scale ranging from 1 (not important) to 5 (very important). These motivations encompassed prosocial and moral incentives such as (1) solidarity with the impoverished and disadvantaged, (2) compassion for those in need, (3) a sense of duty or moral obligation, and (4) religious convictions. Additional items reflect community-oriented and altruistic motives, including (5) assisting disadvantaged individuals, (6) contributing to the local community, (7) fostering social or political change, and (8) acting for social reasons. The survey further evaluates more personal or instrumental motives, such as (9) acquiring new skills and experience, (10) personal satisfaction, (11) availability of free time, and (12) the opportunity to give back. Moreover, the items include (13) identification with individuals suffering, and (14) a more constrained motivation, such as volunteering out of obligation rather than desire, due to perceived inability to refuse. Collectively, these items provide a multidimensional perspective on the determinants influencing voluntary participation within diverse cultural and social settings.

We also examined various demographic factors, including gender, age, and race, which have been shown to significantly influence an individual’s propensity to volunteer. These factors comprised age, categorized into six groups: 15–34, 35–54, and 55 years and older; gender; educational attainment, divided into three levels: low (primary education or below), medium (secondary education), and high (tertiary education); employment status, classified as employed, unemployed, or out of the labor force (including students, retirees, and homemakers); religious attendance, categorized as frequent (at least once per month) or infrequent; and generalized trust, assessed by distinguishing between high trust (respondents indicating that most people are trustworthy) and low trust (respondents expressing that one must be very cautious).

### 2.2 Ethics and informed consent

All WVS data collection procedures conform to ethical standards for social science research. Participation in the survey is voluntary, and respondents give informed consent before taking part. Interviewers ensure that participants understand the purpose of the study, the nature of the questions they will face, and their right to withdraw at any time without penalty. Data are collected and stored anonymously, with identifying information removed to safeguard confidentiality. For the present study, only de-identified secondary data were utilised, and analyses adhered to relevant ethical guidelines for publicly available survey data.

### 2.3 Latent Class Analysis (LCA)

Latent Class Analysis (LCA) is a model-based clustering methodology within the family of finite mixture models, utilised to identify unobserved (latent) categorical variables that elucidate heterogeneity in multivariate categorical or ordinal measurements (17, 26, 38). LCA adopts a person-centered approach, assigning individuals to mutually exclusive and exhaustive latent classes based on their observed response patterns, employing probabilities.

Consider a sample comprising N subjects, whose motivations for volunteering are assessed using J indicators; these indicators are measured on 5 point a Likert scale treated as categorical for purpose of LCA (16). We denote the observed categorical response for volunteer i on motivation indicator j as $Y_{\mathrm{ij}}$ ; where i ranges from 1 to N , and j ranges from 1 to J . The vector $Y_{i}=Y_{i1},Y_{i2}..,Y_{\mathrm{iJ}}$ ) represents the observed responses for individual i across the J indicators. Furthermore, let $C_{i}$ denote an unobserved latent class variable belonging to the set Ω={1,2,…,K}, where K represents the total number of latent classes. The probability of observing a response pattern $Y_{i}$ for individual i is modeled as a finite mixture.

$$P(Y_{i}=y_{i})=\sum_{k=1}^{K}\pi_{k}\prod_{j=1}^{J}P(Y_{\mathrm{ij}}=y_{\mathrm{ij}}|C_{i}=k)$$

where $\pi_{k}=P(C_{i}=k$ ) is the latent class proportion, with $\sum_{k}\pi_{k}=1$ , and $\pi_{\mathrm{ij}|C}=P(Y_{\mathrm{ij}}=y_{\mathrm{ij}}|C_{i}=k$ indicates the item-response probability for indicator j within class k . This construction entails a critical assumption of conditional independence. The observed indicator responses are statistically independent given class membership. Model parameters are generally estimated using maximum likelihood methods, often via the Expectation–Maximisation (EM) algorithm (27). Posterior class membership probabilities are derived using Bayes’ theorem:

πk∣yi=P(Ci=k∣Yi=yi)=(πk∏j=1JP(Yij=yij∣Ci=k))(∑k=1Kπk∏j=1JP(Yij=yij∣Ci=k))

These posterior probabilities denote probabilistic class assignments, with values approaching 1 indicating a more distinct separation between classes (28). The optimal number of latent classes is determined by comparing model fits across different numbers of classes, utilising criteria such as the Akaike Information Criterion (AIC) and the Bayesian Information Criterion (BIC) (20). Likelihood-ratio tests, along with considerations of interpretability, further aid in establishing the final number of classes. Additionally, some latent class analysis (LCA) studies incorporate covariates to elucidate variations in class membership. In longitudinal research, Latent Transition Analysis models are employed to account for changes in latent class membership over time (29).

## 3 Results

The statistical analysis was conducted in several stages utilising R software (40). Initially, descriptive statistics were calculated to summarise the sample. Missing data on motivational indicators was negligible (<2%) and was handled by listwise deletion.

### 3.1 Descriptive statistics

The final sample comprised 2,605 individuals drawn from the World Values Survey (WVS) dataset. Table 1 presents the distribution of the sample by sociodemographic characteristics. There were slightly more males (55.2%) than females (44.8%). Nearly half of respondents were aged 15–34 years (47.3%), followed by those aged 35–54 years (38.2%), while 14.5% were aged 55 years or older. The majority of respondents were married (64.2%), with just over a quarter single/never married (28.2%), and smaller proportions separated (3.1%), widowed (2.7%), or divorced (1.8%). More than two-thirds of the sample were employed (69.0%), while 31.0% were unemployed. In terms of education, 44.2% had upper-level education, 42.0% had middle-level education, and 13.9% had lower-level education. Respondents were drawn primarily from Nigeria (24.2%) and Brazil (24.0%), followed by China (18.3%), Chile (14.1%), Russia (12.2%), Argentina (4.6%), and Japan (2.7%).

**Table 1:** Sociodemographic characteristics of the analytic sample (*N* = 2,605).

Variable	Category	*n*	%
Sex	Male	1,438	55.2
	Female	1,166	44.8
Age group	15–34 yrs	1,231	47.3
	35–54 yrs	996	38.2
	55 + yrs	378	14.5
Marital status	Married	1,671	64.2
	Single/never married	735	28.2
	Divorced	48	1.8
	Separated	80	3.1
	Widowed	71	2.7
Employment	Employed	1,791	69.0
	Unemployed	805	31.0
Education	Lower	350	13.9
	Middle	1,061	42.0
	Upper	1,116	44.2
Country	Argentina	119	4.6
	Brazil	624	24.0
	Chile	367	14.1
	China	477	18.3
	Japan	70	2.7
	Nigeria	631	24.2
	Russia	317	12.2

^a^Education data were available for a subsample (*n* = 2,527).

### 3.2 Latent class analysis model fit and selection

We identified the optimal number of latent classes by fitting models ranging from one to six classes using multiple criteria (Table 2). Model fit was assessed using the Akaike Information Criterion (AIC) and Bayesian Information Criterion (BIC), with lower values indicating better fit. Given that AIC tends to favor more complex models and may overestimate the number of classes (30), we prioritized BIC, which applies a more substantial penalty for model complexity (31). Classification quality was assessed using entropy, with values closer to 1 indicating better class separation (28). Model selection was further guided by parsimony and substantive interpretability of the identified classes (16, 32).

**Table 2:** Model fit indices for latent class analysis models.

Number of classes (*k*)	AIC	BIC	Log-likelihood	Entropy
1	102,611	102,940	−51,249	–
2	94,917	95,580	−47,346	0.884
3	91,796	92,794	−45,728	0.889
4	90,560	91,893	−45,053	0.851
5	89,719	91,387	−44,576	0.860
6	89,223	91,225	−44,270	0.848

Table 2 shows substantial improvements from the one-class to the two-class model (reductions in AIC and BIC of 7,694 and 7,360, respectively) and from the two-class to the three-class model (reductions of 3,121 and 2,786, respectively). Fitting more classes resulted in very minor reductions; for example, three-to-four-class: AIC and BIC reduced by 1,236 and 901; and for four-to-five-class: AIC and BIC reduced by 841 and 506, with very little reduction for five-to-six-class: AIC reduced by only 496 and BIC by only 162. The reductions in observed values of fit indices suggest that both the three- and four-class models appear reasonable; however, the four-class model is much smaller. Regarding entropy, this was highest for the three-class solution (0.889), indicating superior classification quality compared to models with more classes.

We complemented the selection of the number of classes by assessing the classification of the classes based on the normalised item-response probabilities, shown in Tables 3, 4 for three and four classes, respectively. The three-class models show a clearer, more discriminable pattern of low, moderate, and high motivation across all indicators than the four-class model, which shows less distinct profiles and overlapping response patterns that are more difficult to interpret meaningfully. Thus, although both the three- and four-class model solutions are empirically defensible, the three-class model provides a more parsimonious and substantively interpretable representation of volunteer motivation profiles and was therefore retained for subsequent analyses.

**Table 3:** Normalised probabilities under a 3 class model.

Item	Class 1	Class 2	Class 3
A107: Solidarity with the poor and disadvantaged	0.474	0.740	0.948
A108: Compassion for those in need	0.482	0.742	0.952
A109: Opportunity to repay something	0.416	0.596	0.790
A110: Sense of duty, moral, obligation	0.618	0.780	0.928
A111: Identifying with people who suffer	0.476	0.722	0.942
A112: Time on my hands	0.482	0.720	0.850
A113: Personal satisfaction	0.494	0.564	0.744
A114: Religious belief	0.404	0.494	0.810
A115: Help disadvantaged people	0.478	0.714	0.960
A116: Make a contribution to my local community	0.538	0.716	0.910
A117: Bring about social or political change	0.374	0.618	0.732
A118: For social reasons	0.438	0.660	0.790
A119: Gain new skills and useful experience	0.484	0.760	0.904
A120: Did not want to, but could not refuse	0.306	0.442	0.528

**Table 4:** Normalised probabilities under a 4 class model.

Item	Class 1	Class 2	Class 3	Class 4
A107: Solidarity with the poor and disadvantaged	0.956	0.486	0.916	0.688
A108: Compassion for those in need	0.964	0.488	0.918	0.692
A109: Opportunity to repay something	0.842	0.414	0.714	0.570
A110: Sense of duty, moral, obligation	0.948	0.622	0.894	0.748
A111: Identifying with people who suffer	0.956	0.494	0.900	0.670
A112: Time on my hands	0.888	0.484	0.804	0.694
A113: Personal satisfaction	0.792	0.502	0.668	0.538
A114: Religious belief	0.848	0.416	0.732	0.434
A115: Help disadvantaged people	0.976	0.494	0.914	0.658
A116: Make a contribution to my local community	0.948	0.546	0.854	0.678
A117: Bring about social or political change	0.736	0.382	0.720	0.584
A118: For social reasons	0.824	0.440	0.742	0.640
A119: Gain new skills and useful experience	0.928	0.492	0.864	0.728
A120: Did not want to, but could not refuse	0.544	0.302	0.498	0.434

#### 3.2.1 Motivation profiles and class membership

Volunteers were categorised into three groups based on the intensity of their motivation: approximately one-third exhibited low motivation (31.2%), a comparable proportion demonstrated moderate motivation (32.1%), and slightly more than one-third showed high motivation (36.7%). These groups were distinguished primarily by the relative significance attributed to altruistic, moral, social, religious, and self-directed motivations. Volunteers in the low-motivation cohort (Class 1) attributed relatively low importance to all motivational items, whereas those in the moderate-motivation cohort (Class 2) showed intermediate endorsement across motivations. Conversely, volunteers in the high-motivation group (Class 3) consistently regarded all motivational factors considered as highly significant, reflecting robust and comprehensive engagement across all motivational domains.

### 3.3 Socio-demographic and country differences

Table 5 presents the results of the multinomial logistic regression analysis examining socio-demographic predictors of class membership within the three-class solution, with Class 3 (high motivation) as the reference category. Age and gender were significantly associated with class membership. Individuals aged 35–54 and those aged 55 years and older were less likely to belong to the low-motivation group compared to the high-motivation group (35–54 years: OR = 0.62, 95% CI: 0.48–0.80; 55 + years: OR = 0.59, 95% CI: 0.41–0.84). In contrast, associations with the moderate-motivation group were weaker and not statistically significant (35–54 years: OR = 1.04, 95% CI: 0.75–1.45; 55 + years: OR = 0.71, 95% CI: 0.44–1.15). Similarly, women were less likely than men to belong to the low-motivation group (OR = 0.67, 95% CI: 0.55–0.83), while no significant difference was observed for the moderate-motivation group (OR = 0.87, 95% CI: 0.66–1.14).

**Table 5:** Multinomial logistic regression model results using a three-class structure.

Predicter	Category	Class 1 OR (95% CI)	Class 2 OR (95% CI)
(Intercept)	(Intercept)	1.44 (0.74–2.78)	2.15 (1.08–4.28)
Age groups	35–54 yrs	0.62 (0.48–0.80)	1.04 (0.75–1.45)
	55 + yrs	0.59 (0.41–0.84)	0.71 (0.44–1.15)
Education	Middle	0.88 (0.64–1.20)	0.71 (0.47–1.06)
	Upper	1.44 (1.05–1.98)	1.06 (0.71–1.59)
Gender	Female	0.67 (0.55–0.83)	0.87 (0.66–1.14)
Employment status	Employed	0.92 (0.73–1.17)	0.90 (0.66–1.24)
Marital status	Divorced	1.79 (0.87–3.66)	0.47 (0.13–1.74)
	Separated	0.86 (0.49–1.51)	0.95 (0.46–1.94)
	Widowed	1.02 (0.54–1.92)	0.68 (0.26–1.76)
	Single/never married	1.09 (0.83–1.41)	1.26 (0.88–1.79)
Country	BRA	0.53 (0.30–0.93)	0.08 (0.04–0.14)
	CHL	0.74 (0.41–1.31)	0.20 (0.11–0.34)
	CHN	5.25 (2.88–9.58)	0.77 (0.43–1.38)
	JPN	15.27 (4.18–55.70)	5.40 (1.48–19.64)
	NIG	0.24 (0.13–0.42)	0.12 (0.04–0.12)
	RUS	3.16 (1.71–5.84)	0.65 (0.35–1.18)

Additional socio-demographic factors showed more limited or inconsistent associations. Higher educational attainment was associated with an increased likelihood of belonging to the low-motivation group (OR = 1.44, 95% CI: 1.05–1.98), but not the moderate-motivation group (OR = 1.06, 95% CI: 0.71–1.59). Employment status was not significantly associated with class membership (low motivation: OR = 0.92, 95% CI: 0.73–1.17; moderate motivation: OR = 0.90, 95% CI: 0.66–1.24). Marital status effects were generally weak and imprecise; for instance, divorced individuals showed higher odds of belonging to the low-motivation group (OR = 1.79, 95% CI: 0.87–3.66), but lower odds of being in the moderate-motivation group (OR = 0.47, 95% CI: 0.13–1.74), although these estimates were not statistically significant.

Substantial cross-country differences were observed. Compared to Argentina, volunteers from Brazil and Chile were less likely to belong to the low- or moderate-motivation groups. In contrast, volunteers from China and Japan were more likely to be classified as low-motivated. Volunteers from Nigeria were less likely to belong to either the low- or moderate-motivation groups, while those from Russia were more likely to be in the low-motivation group, with no clear difference observed for the moderate-motivation category.

## 4 Discussion and conclusions

This study investigated the heterogeneity of volunteer motivations using Latent Class Analysis (LCA) on data from 2,605 respondents across seven countries participating in the World Values Survey (1982–2022). Volunteers play an important role in addressing societal needs, strengthening community well-being, and promoting social cohesion. Although volunteering is recognised as being driven by multiple motivations, relatively little is known about how these motivations cluster within individuals and vary across socio-demographic and cultural contexts. By identifying latent subgroups based on altruistic, moral, social, religious, and self-oriented motivations, this study provides new insights into the multidimensional nature of volunteer motivation in a cross-national setting.

The findings indicate that a three-class model provides the most parsimonious and substantively interpretable representation of volunteer motivation, supported by the highest entropy value (0.889) and clear class separation. Volunteers were classified into low- (31.2%), moderate- (32.1%), and high-motivation (36.7%) groups. The low-motivation group consistently assigned low importance to all motivational domains, suggesting weaker or more situational engagement, whereas the high-motivation group reported strong endorsement across all motivational domains, reflecting broad and sustained commitment. The moderate-motivation group occupied an intermediate position across all domains. These findings suggest that volunteer motivations differ primarily in their intensity rather than in qualitatively distinct types. This interpretation is consistent with previous research suggesting that volunteer motivation is better conceptualised as a continuum than as discrete categories (33, 34). Although some studies have identified distinct motivational profiles in specific settings (14, 39) the similarity in the relative pattern of motivations across the three classes suggests that the profiles identified in this study primarily reflect differences in motivational strength.

These findings can be interpreted within the framework of Self-Determination Theory (SDT), which conceptualises motivation as a continuum from controlled to autonomous regulation (9). Volunteers in the high-motivation class are likely to be driven by more autonomous motivations, characterised by intrinsic enjoyment and internalised values. In contrast, those in the low-motivation class may be more influenced by external pressures or situational circumstances, reflecting controlled motivation. Volunteers in the moderate-motivation group appear to be motivated by a combination of intrinsic and extrinsic factors. Interpreting the findings through the lens of SDT highlights that volunteer engagement depends not only on the presence of motivation but also on its intensity and degree of internalisation.

Several socio-demographic characteristics were associated with motivational intensity, broadly consistent with previous research. Older volunteers (35–54 years and 55 years and older) were less likely to belong to the low-motivation group, supporting evidence that prosocial engagement tends to strengthen with age (33). Women were also less likely than men to be classified in the low-motivation group, consistent with studies reporting stronger altruistic and social motivations among female volunteers (33, 37). Higher educational attainment was associated with a lower likelihood of belonging to the low-motivation group, reflecting mixed evidence on the relationship between education and volunteering (15, 34). Employment and marital status showed relatively weak or inconsistent associations with motivational profiles, consistent with previous findings (34, 36). Significant cross-national differences were also observed. Volunteers from China and Japan were more likely to belong to the low-motivation group, whereas volunteers from Brazil and Nigeria were less likely to belong to either the low- or moderate-motivation groups. These findings reinforce evidence that cultural norms, civic traditions, and institutional contexts shape volunteer motivations and patterns of engagement (13, 25).

The findings have important implications for volunteer policy and programme development. Recognising that volunteer motivations differ primarily in intensity suggests that recruitment and retention strategies should be tailored to distinct motivational profiles. Volunteers with stronger motivations may benefit from meaningful roles, opportunities for personal development, and recognition of their contributions, whereas those with lower motivational intensity may be encouraged through flexible volunteering opportunities, supportive social environments, and targeted engagement strategies. Taking motivational intensity into account when designing volunteer programmes may improve recruitment, retention, and the overall effectiveness of volunteer initiatives across diverse settings.

This study has several limitations. First, the cross-sectional design precludes causal inference and prevents examination of changes in volunteer motivation over time. Second, the analysis relied on self-reported measures, which may be subject to recall and social desirability biases. Third, although the World Values Survey provides a valuable cross-national dataset, only seven countries were included, limiting the generalisability of the findings. In addition, the conditional independence assumption underlying LCA may not be fully met, and interpreting latent classes inevitably involves some degree of subjectivity. Future studies should consider longitudinal designs, include a broader range of countries, and explore alternative person-centred modelling approaches.

In conclusion, this study demonstrates that volunteer motivation is best understood as a continuum of motivational intensity rather than as distinct motivational types. A three-class solution provided a parsimonious and interpretable representation of volunteer motivation, with socio-demographic and cultural factors influencing membership of the identified motivational profiles. These findings enhance understanding of the heterogeneity of volunteer motivation and provide evidence to inform more targeted recruitment, improved volunteer retention, and the design of volunteer programmes responsive to diverse motivational needs across cultural contexts.

## Data Availability

Publicly available datasets were analyzed in this study. This data can be found at: https://www.worldvaluessurvey.org/.
